# Supplementary material for: Learning curve and surgical outcome of robotic assisted colorectal surgery with ERAS program
Source: Sci Rep. 2022 Nov 29;12:20566. doi: 10.1038/s41598-022-24665-w (PMC9709162; doi:10.1038/s41598-022-24665-w)
Supplement: Supplementary file 2 — Supplementary Information 2. [file 41598_2022_24665_MOESM2_ESM.zip › RAL Raw data-Ñ[▒K/087/OP_ñw╝╨░O▒Kñσ.pdf]

正式摘要(電子簽章：000000218012451)

W52 / W52

臺中榮民總醫院

Operation Note

[redacted]

- 手術日期：2021/05/29
- 手術醫師：蔣鋒帆，CR 楊曙榮
- Anesthesia：GE
- Position：Lithotomy
- Aseptic agents：Soap，Betadine Alcohol，Betadine Aqua

Diagnosis

- Pre-operative diagnosis : Malignant tumor of sigmoid colon 20CM FAV
- Post-operative diagnosis : Malignant tumor of D-colon

Surgical Indication

- removal of tumor

Operation

- Robotic left hemicolectomy

Finding

- 1. One 0.2x0.2 cm tumor at D-colon near splenic flexure
  2. There was no visible liver metastasis, no visible peritoneal seeding
  3. Robotic left hemicolectomy with side-to-side anastomosis, GIA 80/3.8*2, re-inforcement with silk: done.
  4. Vessel ligation: left branch of middle colic and left colic vessels.
  5. Mild adhision around omentun and RLQ abdominal wall
  6. Surgiwrap:+

Procedure

- General endotracheal anesthesia was induced and then the patient was placed in lithotomy position. Preoperative antibiotics were given. A Foley catheter and nasogastric tube were placed. The abdomen and perineum was prepped and draped in standard sterile fashion. A small vertical incision was made near the umbilicus and a 8mm trochar was inserted into the peritoneal cavity by open method. Peumoperitoneum was established with CO2 inflation. Robotic scopy was inserted and then another three 8mm trochars were inserted over midline of abdomen. 5mm trochar was made over RLQ. Tatto was noted near the splenic flexure. After identification of IMV, the IMV was held by grasper and the peritoneum overlying the mesentery was then scored with electrocautery. Dissection was done above the Gerota’s fascia and toward the splenic flexure. The left branch of middle colic artery and left colic artery were then identified, ligated with endoclips, and transected. Using electrocautery, omentum was freed from the transverse colon. The colon was then freed from its peritoneal attachments along the line of Toldt proximally from the splenic flexure and distally to the pelvic inlet. Splenic flexure and descending colon were mobilized. A small midline incision was made. The descending colon was delivered into the incision and points of transection were selected proximally and distally. The bowel was divided and then the specimen was removed. The two ends of the bowel were checked and found to be viable, with excellent blood supply. The fat was gently cleared from the terminal 2-3 mm of the bowel ends. The two end of the bowel were brought into apposition and found to lie comfortably without excessive tension. Side-to-side anastomosis was performed with GIA 80/3.8X2. Interrupted 3-0 silk Lambert sutures were used to re-inforcement at outer layer. The anastomosis was checked and found to be intact and widely patent. One surgiwrap was placed. The fascia of midline wound was closed with interrupted sutures of No.1 Vicryl. The trochar and wound was closed with 4-0 Vicryl. The patient tolerated the procedure well and was

- brought to the post-anesthesia care unit in stable condition.
- Estimated blood loss: minimum
- 器械數量: 正確
- 紗布數量: 正確

Image

- PAINT
  - abdomen

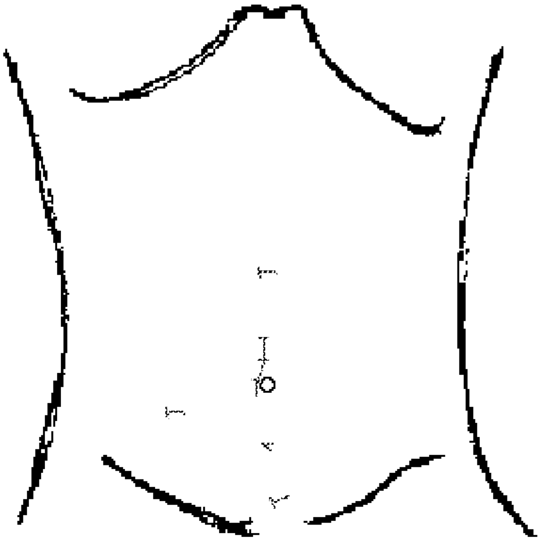

  - colon & rectum

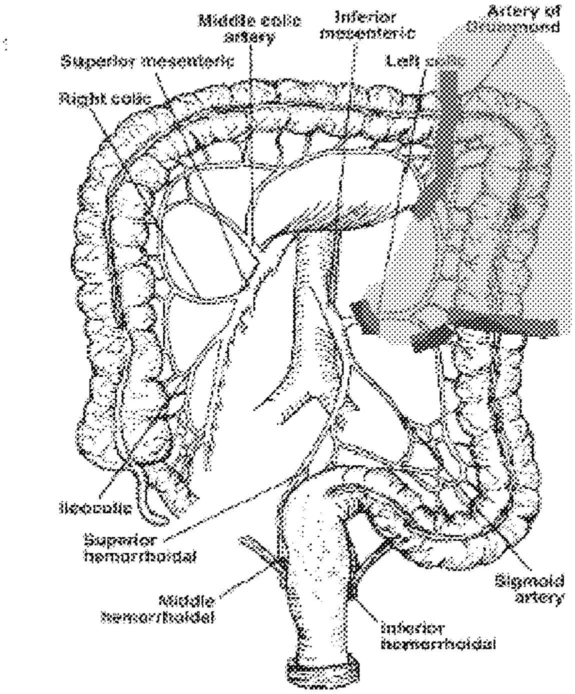

主任 2062F蔣鋒帆 / 醫師 2292G楊曙榮

正式摘要(電子簽章：**000000218012451)**

W52 / W52

doctorRank:B
